# Evolution of Early SARS-CoV-2 and Cross-Coronavirus Immunity

**DOI:** 10.1128/mSphere.00622-20

**Published:** 2020-09-02

**Authors:** Carolin Loos, Caroline Atyeo, Stephanie Fischinger, John Burke, Matthew D. Slein, Hendrik Streeck, Douglas Lauffenburger, Edward T. Ryan, Richelle C. Charles, Galit Alter

**Affiliations:** a Ragon Institute of MGH, MIT, and Harvard, Cambridge, Massachusetts, USA; b Massachusetts Institute of Technology, Cambridge, Massachusetts, USA; c Harvard Virology Program, Boston, Massachusetts, USA; d Virology and Immunology Program, University of Duisburg-Essen, Essen, Germany; e Department of Virology, University Hospital Bonn, Bonn, Germany; f Division of Infectious Disease, Massachusetts General Hospital, Boston, Massachusetts, USA; g Harvard Medical School, Boston, Massachusetts, USA; h Harvard T.H. Chan School of Public Health, Boston, Massachusetts, USA; University of Maryland School of Medicine

**Keywords:** Fc-receptor binding, SARS-CoV-2, antibody response, cross-reactivity

## Abstract

The novel coronavirus, SARS-coronavirus (CoV)-2 (SARS-CoV-2), has caused over 17 million infections in just a few months, with disease manifestations ranging from largely asymptomatic infection to critically severe disease. The remarkable spread and unpredictable disease outcomes continue to challenge management of this infection. Among the hypotheses to explain the heterogeneity of symptoms is the possibility that exposure to other coronaviruses (CoVs), or overall higher capability to develop immunity against respiratory pathogens, may influence the evolution of immunity to SARS-CoV-2. Thus, we profiled the immune response across multiple coronavirus receptor binding domains (RBDs), respiratory viruses, and SARS-CoV-2, to determine whether heterologous immunity to other CoV-RBDs or other infections influenced the evolution of the SARS-CoV-2 humoral immune response. Overall changes in subclass, isotype, and Fc-receptor binding were profiled broadly across a cohort of 43 individuals against different coronaviruses—RBDs of SARS-CoV-2 and the more common HKU1 and NL63 viruses. We found rapid functional evolution of responses to SARS-CoV-2 over time, along with broad but relatively more time-invariant responses to the more common CoVs. Moreover, there was little evidence of correlation between SARS-CoV-2 responses and HKU1, NL63, and respiratory infection (influenza and respiratory syncytial virus) responses. These findings suggest that common viral infections including common CoV immunity, targeting the receptor binding domain involved in viral infection, do not appear to influence the rapid functional evolution of SARS-CoV-2 immunity, and thus should not impact diagnostics or shape vaccine-induced immunity.

**IMPORTANCE** A critical step to ending the spread of the novel severe acute respiratory syndrome coronavirus 2 (SARS-CoV-2) is the ability to detect, diagnose, and understand why some individuals develop mild and others develop severe disease. For example, defining the early evolutionary patterns of humoral immunity to SARS-CoV-2, and whether prevalent coronaviruses or other common infections influence the evolution of immunity, remains poorly understood but could inform diagnostic and vaccine development. Here, we deeply profiled the evolution of SARS-CoV-2 immunity, and how it is influenced by other coinfections. Our data suggest an early and rapid rise in functional humoral immunity in the first 2 weeks of infection across antigen-specific targets, which is negligibly influenced by cross-reactivity to additional common coronaviruses or common respiratory infections. These data suggest that preexisting receptor binding domain-specific immunity does not influence or bias the evolution of immunity to SARS-CoV-2 and should have negligible influence on shaping diagnostic or vaccine-induced immunity.

## Abstract (precis)

A critical step to ending the spread of the novel severe acute respiratory syndrome coronavirus 2 (SARS-CoV-2) is the ability to detect, diagnose, and understand why some individuals develop mild and others develop severe disease. For example, defining the early evolutionary patterns of humoral immunity to SARS-CoV-2, and whether prevalent coronaviruses or other common infections influence the evolution of immunity, remains poorly understood but could inform diagnostic and vaccine development. Here, we deeply profiled the evolution of SARS-CoV-2 immunity, and how it is influenced by other coinfections. Our data suggest an early and rapid rise in functional humoral immunity in the first 2 weeks of infection across antigen-specific targets, which is negligibly influenced by cross-reactivity to additional common coronaviruses or common respiratory infections. These data suggest that preexisting receptor binding domain-specific immunity does not influence or bias the evolution of immunity to SARS-CoV-2 and should have negligible influence on shaping diagnostic or vaccine-induced immunity.

## INTRODUCTION

The SARS-CoV-2 pandemic continues to burden the health care system and has had a major impact on the global economy and social dynamics. While coronaviruses (CoVs) have entered into the human population repeatedly over the past century (1), severe outbreaks with this family of viruses are rare, with reported outbreaks in 2002 caused by the severe acute respiratory syndrome coronavirus (SARS-CoV-1) (2) and in 2012 caused by the Middle East respiratory syndrome coronavirus (MERS-CoV), resulting in 10% and 40% mortality, respectively (3). While SARS-CoV-2 infection appears to be less lethal than SARS-CoV-1 and MERS-CoV, the absence of detailed data on the rate of asymptomatic or mild infections, which do not prompt medical attention, has complicated a true comparison of mortality rates. Well-documented transmission by pre- or asymptomatically infected humans has contributed to the remarkable speed and often uncontrollable spread of SARS-CoV-2. In addition to these lethal CoVs, other CoVs, such as HKU1 and NL63, cause milder influenza virus-like disease (1, 4). Despite their broad prevalence, repeated infections occur with these CoVs over life, hypothesized to occur due to the poor durability of the immune response to these viruses (5). However, despite the lack of seasonal protection against common CoVs, speculations have arisen related to the potential role of cross-CoV immunity in shaping the response to SARS-CoV-2.

SARS-CoV-1 and SARS-CoV-2 share 74% identity, even in the receptor binding domain (RBD), the most variable part of the coronavirus genome (2, 6). In contrast, other coronaviruses such as the NL63 RBD exhibit only 20% sequence identity and HKU1 harbors 2% sequence identity with the SARS-CoV-2 RBD (7). Thus, this limited sequence identity suggests little potential cross-reactivity between the sequences. However, given the increasing possible influence of preexisting cross-reactive antibodies on potential protection or disease enhancement (8), here we aimed to deeply profile and determine whether previous common CoV immunity shapes the evolution of the response to SARS-CoV-2. Both levels of subclasses/isotypes and Fc-receptor binding profiles were interrogated across the RBDs of several CoVs, capturing overall levels and recent inflammatory status of the humoral immune responses. The study highlights the rapid evolution of a robust and highly functional humoral immune response to SARS-CoV-2. However, common CoV RBD-specific immunity appears to have limited to no impact on shaping the SARS-CoV-2 response.

## RESULTS

### Dissecting the early evolution of SARS-CoV-2 humoral immunity.

**FIG 1:**
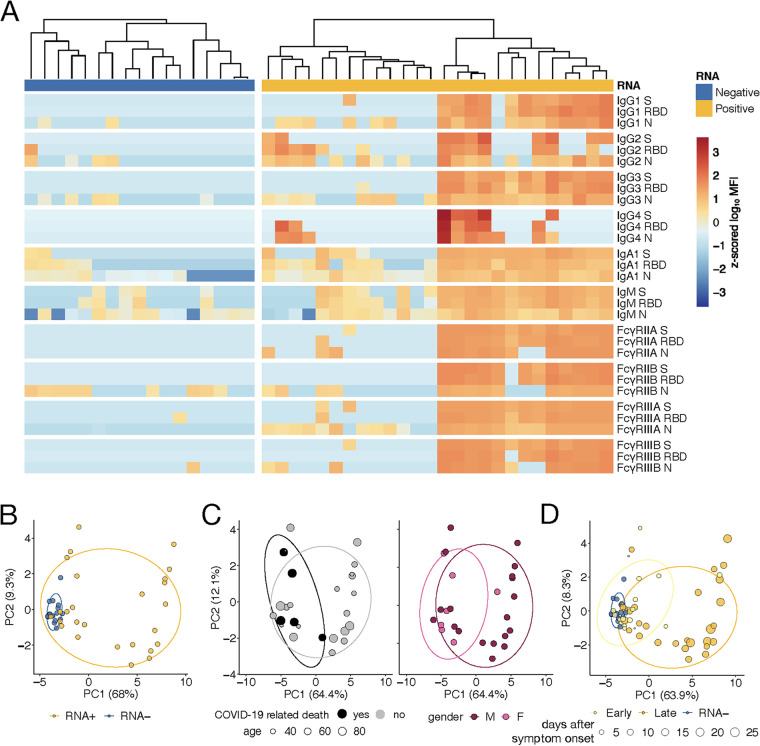
Antibody responses to SARS-CoV-2 S, RBD, and N antigens. (A) The heatmap shows the antibody responses and Fc-receptor binding to SARS-CoV-2 antigens RBD, S, and N. Each row of the heatmap corresponds to Fc-array features while columns correspond to samples from 43 individuals. The annotation row indicates whether patients are SARS-CoV-2 RNA^−^ or RNA^+^. Values were background (2 × PBS) subtracted, log_10_ transformed, and z-scored. High responses are shown in red and low responses in blue. For patients for whom multiple time points are available, the latest time point after symptom onset is shown. (B to D) Principal-component analysis (PCA) for all 43 individuals, using the latest time point for the RNA^+^ individuals (B), the 26 RNA^+^ individuals (C), and all 65 samples points for all 43 individuals (D). Score plots of the first two components are shown, and each point is color coded according to their belonging to different groups. Ellipses show the 70% confidence region for each group assuming a multivariate *t*-distribution.

In order to decipher the humoral immune response to SARS-CoV-2 and cross-reactivity to other common coronaviruses, quantitative data for a cross-sectional sample set of 43 individuals captured at variable time points after symptom onset and hospitalization were generated and analyzed. The heatmap (Fig. 1A) displays the immune responses to SARS-CoV-2 spike (S), nucleocapsid (N), and the spike receptor binding domain (RBD) across subjects, with lower antibody reactivity in non-SARS-CoV-2-infected individuals. To gain a deeper multidimensional analysis of the data, principal-component analysis (PCA) showed expected distinct antibody profiles among RNA^−^ and RNA^+^ individuals (Fig. 1B), where individuals were considered to be RNA^−^ if they had a negative nasopharyngeal (NP) swab PCR test. Within the RNA^+^ individuals, individuals who passed away due to coronavirus disease (COVID-19) were generally older (Fig. 1C). While limited differences were observed between female and male participants, samples from RNA^+^ individuals early following symptom onset (up to 6 days) clustered with RNA^−^ individuals, whereas antibody responses clearly evolved in samples drawn more than 6 days from symptom onset (Fig. 1D).

**FIG 2:**
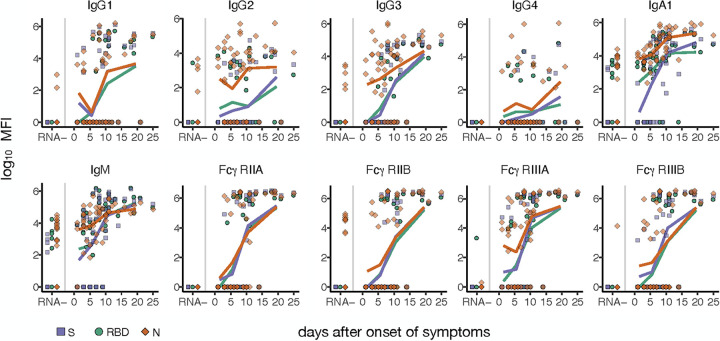
Temporal evolution of the humoral immune response to SARS-CoV-2. The dot plots show antibody titers and FcR binding for SARS-CoV-2 RNA^−^ individuals (left) and values plotted by days after symptom onset for SARS-CoV-2 RNA^+^ individuals (right). Different colors/shapes indicate SARS-CoV-2 antigens S (purple square), RBD (green circle), and N (red diamond). The colored lines depict the mean for SARS-CoV-2 RNA^+^ individuals for each antigen between 0 and 3, 4 and 7, 8 and 13, and 14 and 25 days post-symptom onset. MFI, mean fluorescence intensity.

To explore the kinetics of the evolution of the humoral immune response to SARS-CoV-2, we stratified individuals by time from symptom onset. The evolutions of the S-, N-, and RBD-specific immune responses were compared. Comparable induction of IgG1 responses was observed across all three antigens, emerging in nearly all individuals by day 14 following symptom onset, as has been previously observed (9) (Fig. 2). Similar kinetics were observed for IgG3, an early highly functional antibody subclass (10), particularly for N-specific immunity; these N-specific IgG3 responses appeared to track with background cross-reactive IgG3 responses. More erratic and inconsistent IgG2 and IgG4 responses were observed across the population, albeit more robustly to N, as expected given that these antibody subclasses are less functional and largely selected in the context of nonviral disease (11). Conversely, despite some baseline cross-reactivity in RNA^−^ individuals, robust IgA1 and IgM evolution was observed across antigens (12). Interestingly, IgA1 and IgM responses seem to have emerged synchronously and slightly earlier than IgG1 responses, capturing all infected individuals by day 10, highlighting the unusual class-switching and potential utility of these isotypes in early detection.

Beyond isotype/subclass detection, Fcγ-receptor (FcγR) binding represents a marker of induction of highly functional and proinflammatory antibodies (13). Rapid evolution of broad FcγR binding antibodies was observed across antigens, with early and highly specific detection of FcγRIIA binding antibodies by day 10 following symptom onset, with no background reactivity. Similar profiles were observed across the Fc-receptors, despite some low-level background reactivity. An early rise in N-specific FcγR immunity was observed for FcγRIII binding antibodies. Along these lines, a similar early increase in N-specific IgA1, IgM, IgG2, IgG3, and FcγRIII binding was detected compared to RBD and S. Although less clear for IgG1 and FcγRIIA, this early rise in N-specific immunity may be related to the earlier and more abundant expression of nucleocapsid transcripts during viral infection (14).

### Probing the influence of cross-reactivity to RBDs of other CoVs on SARS-CoV-2 humoral evolution.

**FIG 3:**
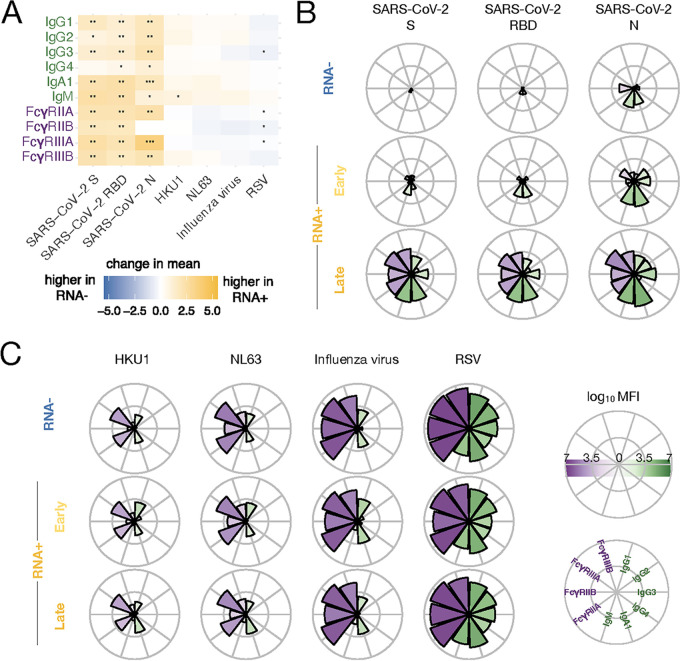
Comparison of SARS-CoV-2 RNA^+^ and RNA^−^ individuals. (A) Heatmap showing the change in mean log_10_ MFI between SARS-CoV-2 RNA^+^ and RNA^−^ samples for different antigens. Blue indicates higher values for RNA^−^ samples, and yellow indicates higher values for RNA^+^ samples. Significance according to Mann-Whitney U test is indicated as * (*q* < 0.05), ** (*q* < 0.01), and *** (*q* < 0.001) for *q* values after Benjamini-Hochberg correction. (B and C) Log_10_ MFI values for SARS-CoV-2 antigens (B) and other antigens (C) for SARS-CoV-2 RNA^−^ (top) and RNA^+^ (bottom) samples, where the positive samples are further divided with respect to the onset of symptoms. Early (middle) samples are taken within the first 6 days after onset of symptoms, and late (bottom) are taken afterward. Higher values are indicated by the size and color of wedges. Fc-receptor binding affinities are shown in purple and antibody subclass/isotype titers in green.

The presence of low-level IgM and IgA1 SARS-CoV-2 binding among RNA^−^ individuals pointed to either potential cross-reactivity to other common CoVs or preexistence of immunity among these early and mucosal responses (15). Given the relatively high seroprevalence of common CoVs, questions have been raised related to the potential influence of these responses on the overall trajectory and quality of the humoral immune response to SARS-CoV-2. Despite the mild sequence identity among the common CoVs, we next compared the overall humoral profiles across common CoV-RBDs (HKU1 and NL63) and other respiratory viruses (influenza virus and respiratory syncytial virus [RSV]). Thus, we analyzed the differences in antibody titers across this spectrum of antigens between SARS-CoV-2-positive and -negative individuals (Fig. 3A). While there was a clear enrichment of responses to SARS-CoV-2 antigens among SARS-CoV-2-infected individuals, no strong differences were observed in responses to the common CoVs, influenza virus, and RSV between the two groups.

Given the more profound differentiation of SARS-CoV-2-infected individuals by IgA1 and IgM immunity (Fig. 3A), we next aimed to dissect the dynamics of the changes in the response to SARS-CoV-2. Low-level cross-reactivity was observed for IgM, IgA1, and FcγRIIB for the SARS-CoV2 N-specific response (Fig. 3B). Conversely, an expansion of IgA1 and IgM N-specific humoral immunity during the early days of infection (0 to 6 days from symptom onset) was observed, followed by RBD- and then S-specific humoral profiles. This cross-reactivity to N may be explained by the fact that N is highly conserved between coronaviruses (16–18) such that preexisting antibodies specific to the N of coronaviruses may be able to bind to the N of SARS-CoV-2. Yet, the responses increased substantially across all antigens (Fig. 3B). Whether these early responses and trajectories were linked to the potential presence of N-cross-reactivity, where class-switched antibodies may represent a surrogate for a helper T cell response, remains uncertain.

To parse the potential influence of cross-CoV-reactivity on shaping the trajectory of the SARS-CoV-2-specific response, the overall profile of reactivity was probed against HKU1, NL63, influenza virus, and RSV (Fig. 3C). A highly synchronized IgG, IgA1, and IgM response was noted across individuals, linked to robust evolution of Fcγ-receptor binding profile. Detectable IgG1 and IgA1 responses were noted to both common CoVs, linked to robust FcγRIIA and FcγRIIIA binding antibodies. Similar IgG1 and IgA1 responses were observed to influenza virus, associated with more functional FcγR binding profiles. Conversely, a broader antibody subclass/isotype and FcγR binding profile was seen for RSV. However, importantly, notable differences were not found in the overall response profile to any of these pathogens across non-SARS-CoV-2-infected, early SARS-CoV-2-infected, or later SARS-CoV-2-infected individuals, which should expand in synchrony to SARS-CoV-2 immunity if cross-reactive. Due to the lack of substantive profile changes across these pathogens with SARS-CoV-2 infection, these data argue for limited cross-reactivity or influence across the responses.

**FIG 4:**
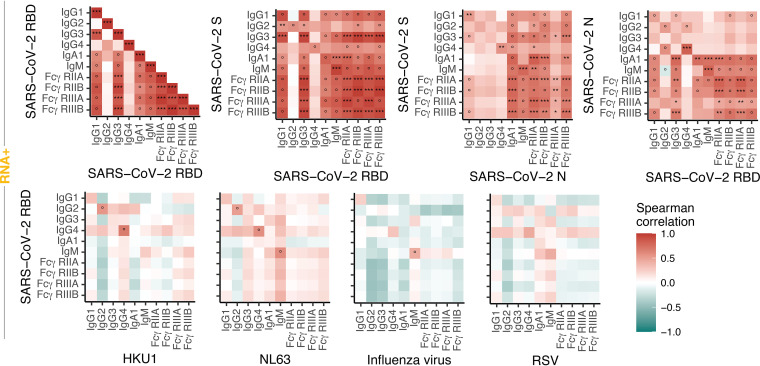
Correlation analysis of SARS-CoV-2 and other antigens. Spearman correlation coefficients for pairwise comparisons of SARS-CoV-2 antigens (upper row) and comparison of SARS-CoV-2 RBD-specific measurements with other antigens (lower row). Significance is indicated as * (*q* < 0.05), ** (*q* < 0.01), and *** (*q* < 0.001) for *q* values after Benjamini-Hochberg correction. The symbol ° indicates that the correlation is driven by values below background (i.e., set to 0) and that the *q* value is >0.05 when removing the samples with value 0. For the individuals with multiple time points, the average levels across time points were used.

We next studied the relationship between SARS-CoV-2 immunity across antigens and across other pathogens (Fig. 4). Strong correlations were observed across the SARS-CoV-2 antigens, highlighting the coordinated induction of highly functional immunity, across isotype/subclass/Fc-receptor binding, to the RBD, S, and N antigens. While some positive relationships were observed among the IgG2, IgG4, and IgM response to the common CoVs and SARS-CoV-2 RBD-specific immunity, most relationships between the SARS-CoV-2 response and common CoVs, influenza virus, and RSV were largely driven by individuals exhibiting low to undetectable titers. Overall, these data suggest that preexisting CoV RBD-specific and other pathogen immunity plays a limited role in shaping SARS-CoV-2 humoral immune responses.

## DISCUSSION

The recent outbreak of SARS-CoV-2 has altered the globe due to its unprecedented speed of dissemination. Treatment of infection has been hampered by our lack of knowledge related to the underlying mechanisms that drive heterogeneous disease outcomes. While the majority of individuals appear to experience mild disease, it remains unclear why a fraction of those infected go on to develop severe and lethal disease. Comorbidities including obesity, heart disease, etc., have been clearly linked to poor disease outcomes (19). However, given the prevalence of other CoVs in the population, hypotheses have emerged related to the potential for cross-CoV immunity. Yet, little is known about the prevalence of CoV-specific immunity at a population level and how it may influence the evolution of SARS-CoV-2 immunity. Similar to previous reports, we observed the development of robust virus-specific IgG, IgM, and IgA1 responses within the first 2 weeks of symptoms (20). This humoral evolution was marked by the rapid evolution of Fc-receptor binding antibodies (Fig. 2 and 3A), highlighting the functional nature of the humoral immune response to SARS-CoV-2. In contrast, the humoral response to the RBDs of more common CoVs was equivalent across SARS-CoV-2 RNA^+^ and RNA^−^ individuals and did not shift with the evolution of infection. While unlikely, based on clinical presentation, it is possible that some of the SARS-CoV-2 RNA^−^ individuals were infected but were not captured by the PCR. However, if this is the case, these individuals would likely be early in their infection course, based on their humoral profile, with low antibody levels similar to the SARS-CoV-2 RNA^+^ individuals within the first 6 days (Fig. 1A). Given the lack of sequence similarity across SARS-CoV-2 and the common CoVs, these data point to the limited influence of cross-CoV immunity on shaping SARS-CoV-2 responses.

HKU1 and NL63 share 20%/26% and 2%/19% similarity with SARS-CoV-2 (RBD/S) (7), respectively, are structurally remarkably distinct, and are therefore unlikely to contribute strongly to cross-reactivity. However, due to enhanced similarity in other genes, including the nucleocapsid, cross-protective immunity may emerge not only at the level of antibody cross-reactivity. Specifically, the presence of cross-reactive T cell immunity (21, 22), targeting conserved linear regions of the virus, could preexist and support the more rapid selection and boosting of humoral immune responses that could then drive enhanced control/protection against SARS-CoV-2. However, if T cell boosting could propagate more effective SARS-CoV-2 immune responses, a shift in the original CoV-humoral immune profile might be observable. No changes were observed in CoV immunity, other than SARS-CoV-2 responses, highlighting the remarkably restricted evolution of the humoral immune response to this CoV alone. Yet, here we used only the RBD from several CoVs, due to its immunologic importance in neutralizing antibody-mediated blockade of infection, which is likely to be key to cross-CoV immunity. Instead, cross-CoV immunity may emerge outside the RBD and happen in a genus-restricted manner potentially explained by conservation of the S2 subunit (23). Further analysis may be required to rule out the possibility of the influence of cross-reactivity on shaping SARS-CoV-2 immunity.

Beyond the potential role of cross-CoV immunity in shaping the initial response to the virus, it is plausible that cross-CoV responses could evolve following SARS-CoV-2 infection to more similar CoVs, such as SARS-CoV-1 (70% identity) and MERS-CoV (50% identity) (24) These similarities may translate to the development of cross-reactive neutralizing antibodies (25). Recent studies have demonstrated that neutralizing antibodies develop in most individuals and seem to be biomarkers of disease progression, with higher neutralizing antibody levels in older individuals and individuals with more severe disease (26). Beyond neutralization, antibodies can also drive innate immune functions, including antibody-dependent cellular cytotoxicity (ADCC), by binding to FcRs which appear to be more sensitive at picking up infection. Here, we find that SARS-CoV-2-specific FcγR binding emerged rapidly following symptom onset, potentially emerging as a more sensitive marker of infection. Therefore, since antibody functions are correlated with FcγR binding, it is likely that individuals induce functional antibodies early in infection. Future studies should explore the timing of functional antibody induction and whether certain antibody functions are important for clearance of SARS-CoV-2 infection. Moreover, in recent nonhuman primate rechallenge and vaccine studies, neutralizing and functional antibodies to RBD and S were shown to predict protection (27, 28), indicating an important immunological role of these antigens in immune protection.

Slowing the spread of the SARS-CoV-2 pandemic will require widespread immune testing and the development of a vaccine. Since antibodies against SARS-CoV-2 N seem to arise earlier than antibodies against RBD and S, N-specific responses may provide earlier diagnostic value. However, some cross-reactivity to N, in RNA^−^ individuals, may render these responses less reliable. However, together N and S/RBD immunity may help guide early diagnosis.

While high-quality and precise serological assays have now emerged, defining the potential influence of cross-CoV immunity on assay performance but also with respect to potential cross-immunity is of utmost importance. However, due to low sequence identity between SARS-CoV-2 and more common CoVs, the data reported here point to limited cross-CoV overlap in humoral responses. Additionally, the lack of relationship between other respiratory pathogens and SARS-CoV-2 additionally suggests that no intrinsic biases exist between the abilities to mount immunity to respiratory pathogens. Thus, although preexisting immunity or enhanced respiratory immunity has been postulated to potentially lower peak responses and shorten durability (29), the data presented here argue that preexisting immunity does not influence SARS-CoV-2 and thus should not influence diagnostics or vaccine-induced immunity. Given the similarity between SARS-CoV-1 and the possibility for future bat-derived CoVs, a multivalent vaccine able to drive immunity to distinct RBDs may ultimately be necessary to protect against different CoV pathogens. Therefore, further research into cross-reactivity of antibodies, especially postvaccination, is needed to decipher the humoral protective immune profile needed not only to end this pandemic but also to prevent future outbreaks caused by CoVs.

## MATERIALS AND METHODS

### Sample set.

Blood samples from SARS-CoV-2 RNA^+^ (*n* = 26) and RNA^−^ (*n* = 17) individuals who were admitted to Massachusetts General Hospital were collected in this study between 13 March 2020 and 31 March 2020. Clinical information on their disease outcome (deceased/discharged), gender, age, and symptom onset were collected (see Table S1 in the supplemental material). The RNA^−^ patients had fever and or symptoms consistent with a respiratory viral infection. This research was approved by the Institutional Review Board of Massachusetts General Hospital, IRB approval no. 2007P002451.

10.1128/mSphere.00622-20.1 TABLE S1 Demographics of the hospitalized patient cohort. Download Table S1, TIF file, 0.7 MB. Copyright © 2020 Loos et al. 2020 Loos et al. This content is distributed under the terms of the Creative Commons Attribution 4.0 International license.

### Subclassing and isotyping via Luminex.

In order to quantify the antigen-specific antibody titer per subclass and isotype as well as Fcγ-receptor levels, a customized Luminex subclassing assay was used (30). Due to the sample-sparing and multiplex-able nature of the assay, we used a Luminex assay to capture data on these samples, following the confirmation of Luminex performance to a qualified enzyme-linked immunosorbent assay (ELISA). Fluorescent carboxyl-modified microspheres (Luminex) were coupled with different antigens: SARS-CoV-2 S (kindly provided by Bing Chen), SARS-CoV-2 RBD, CoV-HKU1 RBD (accession no. AY597011, amino acid [aa] residues 310 to 677), CoV-NL63 RBD (accession no. AKT07952, aa residues 481 to 616) (kindly provided by Aaron Schmidt), SARS-CoV-2 N (Aalto Bio Reagents), influenza virus antigen mix [HA(ΔTM)(A/California/04/2009)(H1N1), HA1(B/Massachusetts/2/2012), and HA1(A/Texas/50/2012)(H3N2)—all from ImmuneTech], and RSV postfusion (NIH). The sequences of HKU1 and NL63 RBD were cloned into the pVRC vector with a C-terminal SBP (streptavidin-binding peptide) tag and produced in 293F cells. Luminex bead regions were coupled via covalent N-hydroxysuccinimide (NHS)–ester linkages utilizing EDC [1-ethyl-3-(3-dimethylaminopropyl)carbodiimide hydrochloride] (Thermo Scientific) and sulfo-NHS (Thermo Scientific) according to the manufacturer’s instructions. Beads (1.2 × 10^3^ per Luminex region) were added in Luminex assay buffer containing 0.1% bovine serum albumin (BSA) and 0.05% Tween 20 to each well of a 384-well plate (Greiner Bio-one). Five microliters of diluted plasma samples or phosphate-buffered saline (PBS) for background assessment (FcγR binding and IgG at a 1:500 dilution, other subclasses/isotypes at 1:100) was added in duplicate and incubated for 16 h at 4°C while rocking at 900 rpm. The immune-complexed microspheres were washed six times with 60 μl of Luminex assay buffer with an automated plate washer (Tecan). Phycoerythrin (PE)-coupled IgG1-, IgG2-, IgG3-, IgG4, IgA1-, or IgM-specific detection reagents (Southern Biotech) were added at 1.3 μg/ml in Luminex assay buffer and incubated for 1 h at room temperature while shaking at 900 rpm. The coated beads were then washed and read on an iQue Screener (Intellicyt) using a robotic arm (PAA). Similarly, for the FcγR binding profiles, recombinant FcγRIIA, FcγRIIB, FcγRIIIA, and FcγRIIIB (Duke Protein Production Facility) were biotinylated (Thermo Scientific), conjugated to streptavidin-PE for 10 min (Southern Biotech) in Luminex buffer, and added at 1 μg/ml. Samples were run in duplicate for each secondary detection agent.

### Statistics.

Twice the PBS control was subtracted from each measurement, negative values were set to 0, and subsequently values were log_10_(*x* + 1) transformed and z-scored. For the heatmap in Fig. 1A, each row is a feature and each column corresponds to one blood sample. The columns are clustered using complete linkage clustering within the columns of RNA^−^ and RNA^+^ individuals. The principal-component analysis (PCA) was performed using the R package ‘ropls’. For the comparison of antibody responses for SARS-CoV-2 RNA^+^ and RNA^−^ individuals, Mann-Whitney U tests were performed, and *P* values were corrected for multiple testing using the Benjamini-Hochberg correction. To assess correlations between antigens, we used Spearman rank correlations and *P* values were corrected for multiple testing using the Benjamini-Hochberg correction. We calculated significances for the correlation coefficients using all samples and samples for which both values are above the background. When the correlation was significant only when using all samples, we indicated it with ° in the heatmap in Fig. 1A. For the individuals with multiple time points, we used the mean levels for the difference and correlation analysis shown in Fig. 3A and Fig. 4. All analyses were performed using R version 3.6.1.

10.1128/mSphere.00622-20.2 DATA SET S1 Systems serology data, including antibody titers and FcR binding levels. Download Data Set S1, CSV file, 0.1 MB. Copyright © 2020 Loos et al. 2020 Loos et al. This content is distributed under the terms of the Creative Commons Attribution 4.0 International license.
