# Supplementary figures and images for: Evolution of Early SARS-CoV-2 and Cross-Coronavirus Immunity
Source: mSphere. 2020 Sep 2;5(5):e00622-20. doi: 10.1128/mSphere.00622-20 (PMC7471005; doi:10.1128/mSphere.00622-20)

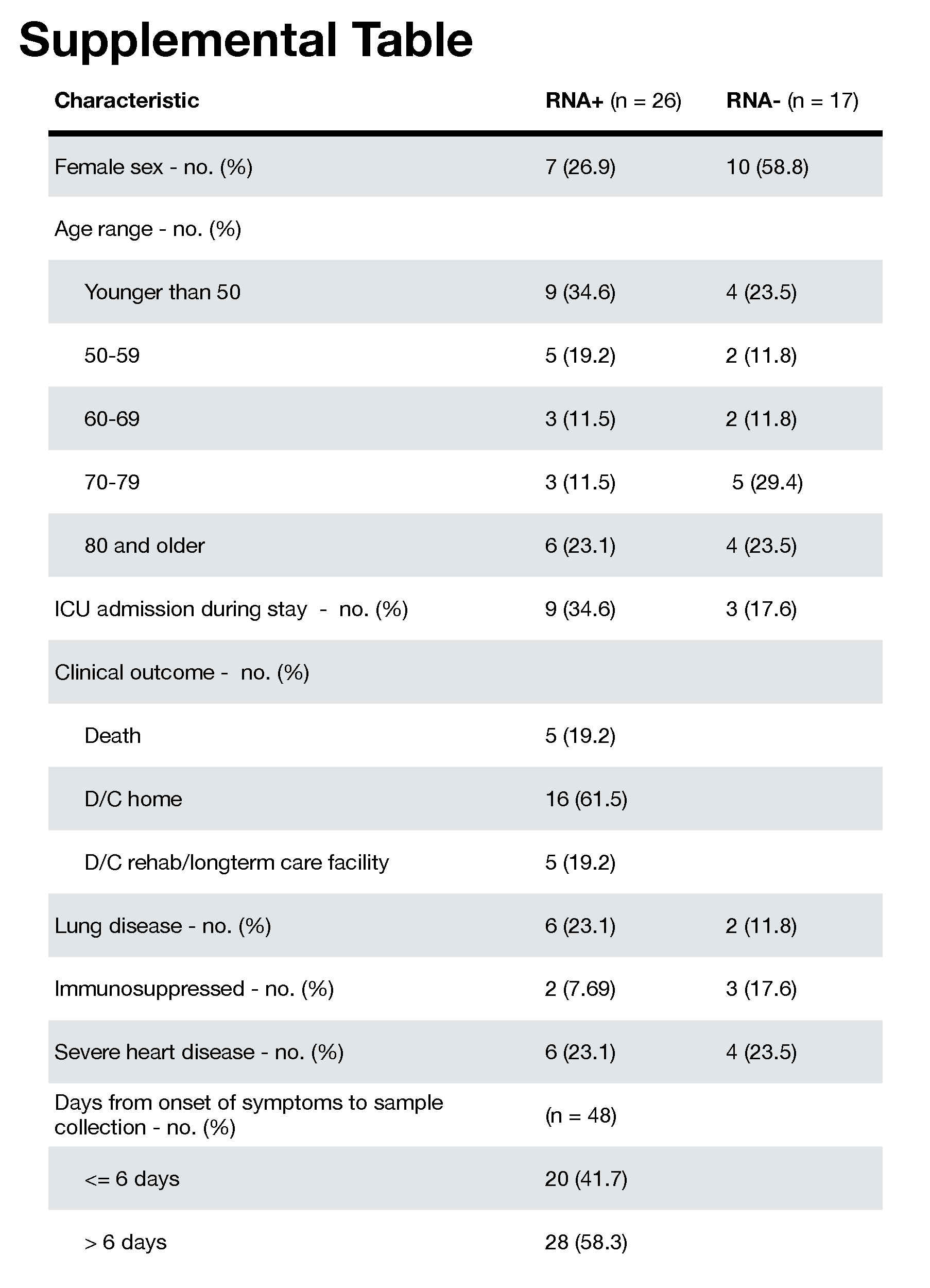

Supplement: TABLE S1 [file mSphere.00622-20-st001.tif]
